# Shall We Play the Same? Pedagogical Perspectives on Infants’ and Children’s Imitation of Musical Gestures

**DOI:** 10.3389/fpsyg.2020.01087

**Published:** 2020-06-03

**Authors:** Manuela Filippa, Maria Grazia Monaci, Susan Young, Didier Grandjean, Gianni Nuti, Jacqueline Nadel

**Affiliations:** ^1^Neuroscience of Emotion and Affective Dynamics Laboratory, Department of Psychology and Educational Sciences, Swiss Center for Affective Sciences, University of Geneva, Geneva, Switzerland; ^2^Division of Development and Growth, Department of Paediatrics, Gynaecology and Obstetrics, University of Geneva, Geneva, Switzerland; ^3^Department of Social Sciences, University of Valle d’Aosta, Aosta, Italy; ^4^University of Roehampton London, London, United Kingdom; ^5^Sorbonne-University, CNRS UMR 7225, Paris, France

**Keywords:** imitation, gesture, action-perception coupling, children, music, sound exploration

## Abstract

Imitation, both gestural and vocal, has been acknowledged to be at the origin of human communication (Donald, 1991). Music is often considered to be the first means of communication of emotion via both vocal and gestural synchronization (Malloch, 1999; Malloch and Trevarthen, 2009). Instrumental music is part of the human heritage for more than 35,000 years before our era (Aimé et al., 2020). However, very little is known about the acquisition of gestures that produce sounds (i.e., musical gestures) and their role in the development of music and musicality. In the present paper, we propose that studying early synchronous imitation of musical gestures is essential both for investigating the development of the early action–perception system and for outlining early music interventions during infancy. We designed double musical objects which can be used in preschool music education for prompting synchronic imitation of musical gestures between adult and child, and between dyads of infants. We conclude by proposing a novel pedagogical perspective in music education for the early years which links the privileged orientation of infants and children towards sound discoveries with the development of perception-action coupling via imitation of musical gestures.

## Introduction

Imitative multimodal abilities have been acknowledged to be fundamental during infancy, for acquiring linguistic, social and emotional competences (Simpson et al., 2014; Kugiumutzakis and Trevarthen, 2015). If vocal imitation is correlated with speech development (Kuhl and Meltzoff, 1996), gestural imitation is also highly predictive of communicative development (Uzgiris, 1972, 1999). Through functional plasticity, early imitation is at the core of both learning and communication experience (Nadel, 2014).

From birth onwards, infants imitate gestures (Field et al., 1982; Meltzoff and Moore, 1983; Soussignan et al., 2011; Meltzoff et al., 2018), and sounds (Kugiumutzakis, 1998) that they have in their motor repertoire. They also imitate the rhythm of a vocal model (Papoušek and Papoušek, 1989), thus underlining that imitation is an interpersonal phenomenon. Within this framework, reciprocal imitations of early vocal interactions in mother-infant dyads are well documented (Kugiumutzakis, 2017). Musical acoustic techniques applied to proto- conversations show how a mother speaking to her infant produces musically organized utterances to which the infant responds synchronously by moving in rhythmic expressive ways (Trevarthen, 2002). Indeed, gestural synchronic imitation is the first format underlying music, very early in infancy (Zentner and Eerola, 2010), as shown by the widespread practice of infant-directed music production such as lullabies and nursery rhymes (Trehub et al., 1993; Trehub, 2000).

Though synchrony is needed to perform any social interaction, a privileged use of synchronic imitation is music: piano duets, orchestral playing, jam sessions, singing together, all require matching one’s rhythm to that of a partner. However, the evolution of young children’s motor imitation during musical play has been poorly investigated.

This perspective paper aims to underline the potentialities of early synchronic imitation of musical gestures; this particular category of gestures that produce sound. As a novel research field, it could first, give new insights into the origins and the development of human musical experience, with specific emphasis on children’s music production with objects and related emotional experience during such practice. Secondly, it can constitute an excellent context for identifying the development of perception-action coupling in music production. Finally, we suggest potential lines of research, both in music education and in music psychology. We propose designs and procedures enabling to elicit the imitation of musical gestures for music education and early therapeutic interventions in infancy.

## Imitation in Early Phases of Development

It is argued that neonatal imitation is a specific form of general imitation abilities that develop throughout the lifespan (Maratos, 1998). Neonatal imitation, although experience-dependent, adapts action to perception with high plasticity. The plasticity of early imitation is of paramount importance for further development as it allows vicarious processes to take place (Nadel, 2014). Interestingly for our specific perspective, newborns imitate not only facial gestures but also precise bodily movements such as finger movements and can match the specific dynamics of an action pattern (Nagy et al., 2005).

The development of imitation in infancy requires the acquisition of a wide range of procedures around the same motor patterns, including vocal patterns, the storage of corresponding sensorimotor feedbacks and motor imagery, and the recall of somatotopic and proprioceptive information related to movement and sound perception (Nadel, 2014). Indeed, imitation enriches an individual’s motor repertoire with gestures and actions that are acquired via watching or listening to other people. Moreover, besides the learning function, an essential function of imitation during infancy is non-verbal communication through synchronic sharing of motor patterns between partners (Nadel, 2014). The interactive emotional engagement during such shared actions producing sounds is likely to help the development of social and emotional abilities. During synchronic imitation, infants co-regulate their actions with an adult (Fogel, 1993) and, from two months on, they take conversational turns by alternating the roles of imitator and model, in a circle of reciprocity (Nadel, 2002). Many studies have demonstrated that infants immediately know if someone is synchronously engaged with them or not (Murray and Trevarthen, 1985; Nadel et al., 1999). In adults, electrophysiological studies reveal that synchrony emerges in the oscillation of brain waves during synchronic imitation (Tognoli et al., 2007; Dumas et al., 2010). When we do the same at the same time, we interact via sharing actions or representations. We share our motor patterns, the most intimate part of ourselves. We generate similar motor outputs that produce common visual and auditory outcomes (Nadel, 2014).

Within this framework, sound appears to play a crucial role in the development of infants’ and children’s ability to match others’ behaviors (Jones, 2009; Piaget, 2013). Although human beings need imitation abilities to synchronize with others during interactions, a privileged context requiring synchronous capacities is music production.

## Music as a Privileged Context for Synchronic Imitation

Moving rhythmically to a musical beat and synchronizing to a proposed rhythm is observed in every human culture (Nettl, 2000) and very early in infancy (Zentner and Eerola, 2010), making it probably a universal musical experience. Synchronic imitation of movements, voices and sounds is one of the key characteristics of music-making during ritual experiences (Dissanayake, 2006). Human orientation and the ability to synchronize to proposed rhythm sequences could represent a cognitive adaptation for music-making (Patel and Iversen, 2006; Cross and Woodruff, 2009; Fitch and Popescu, 2019). Synchronous imitation of rhythms and gestures have positive effects on affiliation processes (Hove and Risen, 2009) and on the increase of prosocial behaviors in infancy (Kirschner and Tomasello, 2010; Cirelli et al., 2014). Peer imitation significantly influences expressive movements in response to music stimulus, in both preschool and kindergarten children (Flohr and Brown, 1979).

It is known that music-making with others is a multimodal activity that engages brain networks, including the mirror neuron system, particularly in the case of sensorimotor synchronization (Repp and Su, 2013). During music performance, where participants were asked to judge the degree of “groove” in the music presentation, sensorimotor coupling strength was significantly associated with higher levels of groove (Tomic and Janata, 2008).

In adults, areas of the brain associated with the processing of reward are active when individuals experience synchrony during joint music-making, and these reward signals are associated with an increase of prosocial behavior toward the music partner (Kokal et al., 2011). It has been shown that subjective entrainment, at both motor and visceral levels, with music tempi and rhythms, is correlated with emotional feelings emerging during music listening (Labbé and Grandjean, 2014). Furthermore, in Wiltermuth and Heath (2009) study, participants engaged in synchronous or asynchronous walking, singing, and hand movement activities. The participants who had synchronized with each other proved to be more cooperative than the others. Whether this is the case also for children, is still unknown. Double musical objects may offer a suitable methodology to document this question.

Even if synchronous imitation is commonly adopted in music learning, its pedagogical applications have just started to be of systematic interest (Buccino et al., 2004). Emerging research, for example, is starting to reflect on the role of mirror neurons in music teaching and learning abilities (e.g., Addessi and Volpe, 2011; Schiavio and Timmers, 2016).

## The Origins of Music Production: the Musical Gesture

In recent years, embodied music cognition has started to reconsider the role and foundations of musical gestures in adults (Godøy and Leman, 2010). From this perspective, music cognition is not just a matter of perception, but involves the whole human body via acting (Varela et al., 2016). Likewise, in infancy, sound perception is linked to the motor action performed to create it. Active experience is critical to modulate motor activity during action perception early in development (Gerson et al., 2015). Among 10-month-old infants the researchers demonstrated a direct, causal effect of the active experience of sound-making gestures on the neural correlates of the same action perception (Gerson et al., 2015).

Similarly, musical gestures match their temporal profiles with the emotion expressed (Juslin and Laukka, 2004) and support universal expressions of emotion (Sievers et al., 2013), as physical and mental parts of “inner life” (Langer, 1957). Music, voice and movement are not only intimately related and share the same dynamic model for expressing emotions (Bänziger et al., 2009; Frühholz et al., 2016), but they are also the basis of human communicative musicality (Malloch, 1999). In the same way, musical gestures can represent a medium to create contact with a patient in therapeutic contexts (Benenzon, 2007).

**FIGURE 1:**
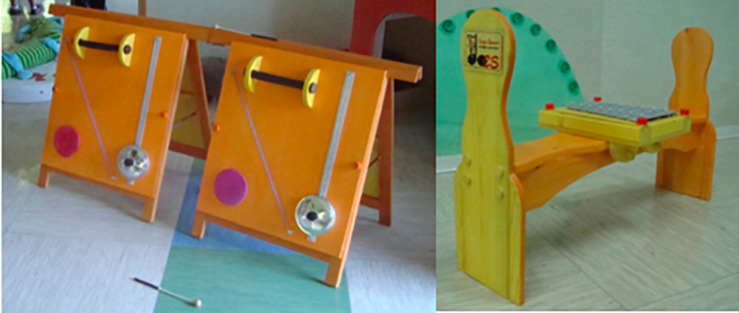
Gives the example of two specific tools which have been tested in kindergarten. Two examples of identical and shared sound objects. Adapted with permission from Nuti and Filippa (2016).

As recently reviewed by Flilippa in a submitted paper, only a few studies that represents Figure 1 have investigated the origin and ontogenesis of musical gestures, and none of them has examined the role of perception-coupling via synchronic imitation in the development of joint music-making.

However, in seminal studies mainly conducted for pedagogical purposes, we can find qualitative descriptions of the acquisition of musical gestures. In Delalande’s descriptions (Delalande, 2015) when infants and young children create sounds by acting on an object like a bunch of keys or a more complex object like a zither or a cymbal, they produce repeated gestures, with simple variations. When a particular sound holds the child’s attention, they stand still for some seconds, and they often gaze at a caring listener, to share this new, significant discovery. This new sound discovery is defined as *trouvaille* (Delalande, 2015; Filippa, 2015). Attracted by this new sound, children concentrate on sound production and they continue to explore it, through repetitions and variations, a fundamental source of learning (Imberty, 2003; Ambrosini et al., 2013). Thus, they develop new musical gestures, and they enlarge their motor repertoire. Using our design with identical double objects, each young partner of a dyad would benefit from the other’s discovery, and would enlarge their motor repertoire by listening and playing the same.

## Imitation With Double Identical Sound Objects

Given that synchrony is the primary vehicle of emotional communication, and that synchronic imitation offers an ideal way to learn and communicate at the same time, we propose to use the procedure designed by Nadel with sound objects. Nadel’s team developed a large series of experiments in order to test the existence of a non-verbal format of communication via synchronic imitation prior to language (see for instance Nadel and Badonnière, 1882; Nadel and Pezé, 1993; Nadel, 2002, 2014). To allow synchrony to spontaneously take place, identical objects were presented to dyads aged 15, 18, 21, 24, and 30 months and triads aged 30, 36, and 42 months. In one study comparing the same dyads aged 24 months when presented with 10 duplicate objects and 20 different objects, it was possible to show, while coding 31.875 s of protocols: that holds of similar objects lasted far longer than holds of different objects (*M* = 31 vs. 13 s); that attention to the partner’s action was far higher with similar objects in the hands (67 vs. 48%); that holds and discards were mostly simultaneous with similar objects (less than one second discard) while they were unrelated with different objects in the hands; and that the partners take turns with high degrees of symmetry between imitate and be imitated. Indeed, in this experimental condition, a genuine communicative system takes place that shows the three features of any communication format: turn-taking, joint attention, and synchrony (Nadel and Pezé, 1993). Since being imitated acts as a nebulization of oxytocin for affiliative behavior in persons with ASD (Delaveau et al., 2015), we can predict that synchronic imitation will impact on affiliative processes, during joint music-making with similar musical objects.

Moreover, we can assume that sound gestures and perception of sounds develop together from the first months of life and that the execution of the sound gesture provides a unique advantage, compared to pure observation, for the sensitivity to multisensory synchrony (Gerson et al., 2015). On this assumption we can predict that the imitation of sound gestures, early in infancy, may represent an essential source for learning and socio-emotional communicative skills. Inspired by the procedure and results, we have designed musical tools likely to elicit synchronic imitation of musical gestures (Filippa et al., 2020).

To conclude this perspective paper we propose that one research area that is particularly well suited for investigating the development of early imitation mechanisms is music, and, more specifically, the imitation of musical gestures by infants. In this context, the development of the early action–perception system as part of children’s learning and communication strategies, is specifically involved.

Interaction with pairs or with adults through the generation of sounds is also characterized by emotional aspects such as emotional resonance, shared feelings and affiliative processes that can help infants to better understand social interactions. It can also be argued that such shared emotional experiences can train infants’ abilities to use emotion for regulating social interactions.

Finally, we propose that eliciting the exploration of musical gestures in infancy can have a significant impact on early childhood music education, restoring the importance of bodily gestures as the origin of human musicality.

## Author Contributions

MF and JN contributed to the conception and design of the manuscript. MF wrote the first draft of the manuscript. MM, SY, DG, and GN wrote sections of the manuscript. All authors contributed to manuscript revision, read and approved the submitted version.

## Conflict of Interest

The authors declare that the research was conducted in the absence of any commercial or financial relationships that could be construed as a potential conflict of interest.

## References

[B1] Addessi A. R. Volpe G. (2011). “The MIROR project,” in *Proceedings of the European Conference on Technology Enhanced Learning*, (Berlin: Springer), 15–28.

[B2] Aimé C. Le Covec M. Bovet D. Esseily R. (2020). La musicalité est-elle un héritage de notre histoire biologique ? *Enfance* 1 41–66.

[B3] Ambrosini E. Reddy V. De Looper A. Costantini M. Lopez B. Sinigaglia C. (2013). Looking ahead: anticipatory gaze and motor ability in infancy. *PLoS One* 8:e67916. 10.1371/journal.pone.0067916 23861832 PMC3701628

[B4] Bänziger T. Grandjean D. Scherer K. R. (2009). Emotion recognition from expressions in face, voice, and body: the Multimodal Emotion Recognition Test (MERT). *Emotion* 9:691. 10.1037/a0017088 19803591

[B5] Benenzon R. O. (2007). The benenzon model. *Nord. J. Music Ther.* 16 148–159. 10.1080/08098130709478185

[B6] Buccino G. Vogt S. Ritzl A. Fink G. R. Zilles K. Freund H. J. (2004). Neural circuits underlying imitation learning of hand actions: an event-related fMRI study. *Neuron* 42 323–334. 10.1016/s0896-6273(04)00181-3 15091346

[B7] Cirelli L. K. Einarson K. M. Trainor L. J. (2014). Interpersonal synchrony increases prosocial behavior in infants. *Dev. Sci.* 17 1003–1011. 10.1111/desc.12193 25513669

[B8] Cross I. Woodruff G. E. (2009). Music as a communicative medium. *Prehist. Lang.* 1 113–144.

[B9] Delalande F. (2015). “Naissance de la Musique. Les Explorations Sonores de la Première Enfance. Rennes: Presses Universitaires de Rennes. Original Edition,” in *La Nascita Della Musica: Esplorazioni sonore Nella Prima Infanzia*, Ed. Delalande F. (Milano: Franco Angeli).

[B10] Delaveau P. Arzounian D. Rotgé J. Y. Nadel J. Fossati P. (2015). Does imitation act as an oxytocin nebulizer in autism spectrum disorder? *Brain* 138 e360–e360. 10.1093/brain/awv060 25797892

[B11] Dissanayake E. (2006). “Ritual and ritualization: musical means of conveying and shaping emotion in humans and other animals,” in *Music and Manipulation: On the Social Uses and Social Control of Music*, eds Brown S. Voglsten U. (Oxford: Berghahn Books), 31–56.

[B12] Donald M. (1991). *Origins of the Modern Mind.* Cambridge: Harvard University Press.

[B13] Dumas G. Nadel J. Soussignan R. Martinerie J. Garnero L. (2010). Inter-brain synchronization during social interaction. *PLoS One* 5:e12166. 10.1371/journal.pone.0012166 20808907 PMC2923151

[B14] Field T. Woodson W. Greenberg R. Cohen C. (1982). Discrimination and imitation of facial expressions by neonates. *Science* 218 179–181. 10.1126/science.7123230 7123230

[B15] Filippa M. (2015). “Explorations prolongées d’une trouvaille,” in *Naissance de la Musique. Les Explorations Sonores de la Première Enfance*, ed. Delalande F. (Rennes: Presses Universitaires de Rennes).

[B16] Filippa M. Cornara S. Monaci M. G. Grandjean D. Nuti G. Nadel J. (2020). L’imitation sonore durant la période préverbale : enjeux théoriques et dispositifs. *Enfance* 1 131–148.

[B17] Fitch W. Popescu T. (2019). The world in a song. *Science* 366 944–945. 10.1126/science.aay2214 31753980

[B18] Flohr J. W. Brown J. (1979). The influence of peer imitation on expressive movement to music. *J. Res. Music Educ.* 27 143–148. 10.2307/3344965

[B19] Fogel A. (1993). “Two principles of communication: co-regulation and framing,” in *New Perspectives in Communicative Development*, eds Nadel J. Camaioni L. (London: Routledge), 9–22. 10.4324/9781315111322-3

[B20] Frühholz S. Trost W. Kotz S. A. (2016). The sound of emotions—Towards a unifying neural network perspective of affective sound processing. *Neurosci. Biobehav. Rev.* 68 96–110. 10.1016/j.neubiorev.2016.05.002 27189782

[B21] Gerson S. A. Bekkering H. Hunnius S. (2015). Short-term motor training, but not observational training, alters neurocognitive mechanisms of action processing in infancy. *J. Cogn. Neurosci.* 27 1207–1214. 10.1162/jocn_a_00774 25514654

[B22] Godøy R. I Leman M. (eds) (2010). *Musical Gestures: Sound, Movement, and Meaning.* Abingdon: Routledge.

[B23] Hove M. J. Risen J. L. (2009). It’s all in the timing: interpersonal synchrony increases affiliation. *Soc. Cogn.* 27 949–960. 10.1521/soco.2009.27.6.949

[B24] Imberty M. (2003). Langage, musique et cognition: quelques remarques sur l’évolution nécessaire des problématiques psychologiques des vingt dernières années. *Circuit Musiques Contemp.* 13, 93–110. 10.1521/soco.2009.27.6.949

[B25] Jones S. S. (2009). The development of imitation in infancy. *Philos. Trans. R. Soc. B* 364 2325–2335. 10.1098/rstb.2009.0045 PMC2865075 19620104

[B26] Juslin P. N. Laukka P. (2004). Expression, perception, and induction of musical emotions: a review and a questionnaire study of everyday listening. *J. New Music Res.* 33 217–238. 10.1080/0929821042000317813

[B27] Kirschner S. Tomasello M. (2010). Joint music making promotes prosocial behavior in 4-year-old children. *Evol. Hum. Behav.* 31 354–364. 10.1016/j.evolhumbehav.2010.04.004

[B28] Kokal I. Engel A. Kirschner S. Keysers C. (2011). Synchronized drumming enhances activity in the caudate and facilitates prosocial commitment-if the rhythm comes easily. *PLoS One* 6:e27272. 10.1371/journal.pone.0027272 22110623 PMC3217964

[B29] Kugiumutzakis G. (1998). “Neonatal imitation in the intersubjective companion space,” in *Intersubjective Communication and Emotion in Early Ontogeny*, ed. Bräten S. (Cambridge: Cambridge University Press), 63–88.

[B30] Kugiumutzakis G. (2017). “Intersubjective vocal imitation in early mother-infant interaction,” in *New Perspectives in Early Communicative Development*, eds Nadel J. Camaioni L. (Londres: Routledge), 23–47. 10.4324/9781315111322-4

[B31] Kugiumutzakis G. Trevarthen C. (2015). “Neonatal imitation,” in *International Encyclopedia of the Social & Behavioral Sciences*, 2nd Edn, Vol. 16 ed. Wright J. D. (Oxford: Elsevier), 481–488. 10.1016/b978-0-08-097086-8.23160-7

[B32] Kuhl P. K. Meltzoff A. N. (1996). Infant vocalizations in response to speech: vocal imitation and developmental change. *J. Acoust. Soc. Am.* 100 2425–2438. 10.1121/1.417951 8865648 PMC3651031

[B33] Labbé C. Grandjean D. (2014). Musical emotions predicted by feelings of entrainment. *Music Percept.* 32 170–185. 10.1525/mp.2014.32.2.170

[B34] Langer S. K. K. (1957). *Philosophy in a New Key: A Study in the Symbolism of Reason, Rite, and Art.* Cambridge, MA: Harvard University Press.

[B35] Malloch S. Trevarthen C. (eds) (2009). *Communicative Musicality: Exploring the Basis of Human Companionship.* Oxford: Oxford University Press.

[B36] Malloch S. N. (1999). Mothers and infants and communicative musicality. *Musicae Sci.* 3 29–57. 10.1177/10298649000030s104

[B37] Maratos O. (1998). “Neonatal, early and later imitation: same order phenomena?,” in *The Development of Sensory, Motor and Cognitive Capacities in Early Infancy: From Perception to Cognition*, eds Simion F. Butterworth G. (Hove: Psychology Press Ltd), 145–160.

[B38] Meltzoff A. N. Moore M. K. (1983). Newborn infants imitate adult facial gestures. *Child Dev.* 54 702–709. 10.2307/1130058 6851717

[B39] Meltzoff A. N. Murray L. Simpson E. Heimann M. Nagy E. Nadel J. (2018). Re-examination of Oostenbroek et al.(2016): evidence for neonatal imitation of tongue protrusion. *Dev. Sci.* 21:e12609. 10.1111/desc.12609 28952202 PMC6710010

[B40] Murray L. Trevarthen C. (1985). “Emotional regulation of interactions between 2-month-olds and their mothers,” in *Social Perception in Infants*, eds Field T. Fox N. (Norwood, NJ: Ablex Publishers), 177–197.

[B41] Nadel J. (2002). “Imitation and imitation recognition: their social use in healthy infants and children with autism,” in *The Imitative Mind: Development, Evolution and Brain Bases*, eds Meltzoff A. N. Prinz W. (Cambridge: Cambridge University Press), 42–62. 10.1017/cbo9780511489969.003

[B42] Nadel J. (2014). *How Imitation Boosts Development in Infancy and Autism Spectrum Disorder.* Oxford: Oxford University Press.

[B43] Nadel J. Badonnière P. M. (1882). The social function of reciprocal imitation in 2-year-old peers. *Int. J. Behav. Dev.* 5 95–109. 10.1177/016502548200500105 11944873

[B44] Nadel J. Carchon I. Kervella C. Marcelli D. Réserbat-Plantey D. (1999). Expectancies for social contingency in 2-month-olds. *Dev. Sci.* 1 164–174.

[B45] Nadel J. Pezé A. (1993). “What makes immediate imitation communicative in toddlers and autistic children?,” in *New Perspectives in Early Communicative Development*, eds Nadel J. Camaioni L. (London, NY: Routledge), 139–156. 10.4324/9781315111322-9

[B46] Nagy E. Compagne H. Orvos H. Pal A. Molnar P. Jansky I. (2005). Index finger movement imitation by human neonates. *Pediatr. Res.* 8 749–753. 10.1203/01.PDR.0000180570.28111.D9 16189204

[B47] Nettl B. (2000). “An ethnomusicologist contemplates universals in musical sound and musical culture,” in *The Origins of Music*, eds Wallin N. L. Merker B. Brown S. (Cambridge, MA: MIT Press), 463–472.

[B48] Nuti G. Filippa M. (2016). *un nido di suoni.* Firenze: ed. Polistampa.

[B49] Papoušek M. Papoušek H. (1989). Forms and functions of vocal matchingin interactions between mothers and their pre-canonical infants. *First Lang.* 9 137–158.

[B50] Patel A. D. Iversen J. R. (2006). “A non-human animal can drum a steady beat on a musical instrument,” in *Proceedings of the 9th International Conference on Music Perception & Cognition (ICMPC9)*, eds Baroni M. Addessi A. R. Caterina R. Costa M. (Bologna: The Society for Music Perception & Cognition and European Society for the Cognitive Sciences of Music), 477.

[B51] Piaget J. (2013). *Play, Dreams and Imitation in Childhood.* Abingdon: Routledge.

[B52] Repp B. H. Su Y. H. (2013). Sensorimotor synchronization: a review of recent research (2006–2012). *Psychonom. Bull. Rev.* 20 403–452. 10.3758/s13423-012-0371-2 23397235

[B53] Schiavio A. Timmers R. (2016). Motor and audiovisual learning consolidate auditory memory of tonally ambiguous melodies. *Music Percept.* 34 21–32. 10.1525/mp.2016.34.1.21

[B54] Sievers B. Polansky L. Casey M. Wheatley T. (2013). Music and movement share a dynamic structure that supports universal expressions of emotion. *Proc. Natl. Acad. Sci. U.S.A.* 110 70–75. 10.1073/pnas.1209023110 23248314 PMC3538264

[B55] Simpson E. A. Murray L. Paukner A. Ferrari P. F. (2014). The mirror neuron system as revealed through neonatal imitation: presence from birth, predictive power and evidence of plasticity. *Philos. Trans. R. Soc. B* 369:1644. 10.1098/rstb.2013.0289 24778381 PMC4006187

[B56] Soussignan R. Courtial A. Canet P. Danon-Apter G. Nadel J. (2011). Human newborns match tongue protrusion of disembodied human and robotic mouths. *Dev. Sci.* 14 385–394. 10.1111/j.1467-7687.2010.00984.x 22213907

[B57] Tognoli E. Lagarde J. DeGuzman G. C. Kelso J. S. (2007). The phi complex as a neuromarker of human social coordination. *Proc. Natl. Acad. Sci U.S.A.* 104 8190–8195. 10.1073/pnas.0611453104 17470821 PMC1859993

[B58] Tomic S. T. Janata P. (2008). Beyond the beat: modeling metric structure in music and performance. *J. Acoust. Soc. Am.* 124 4024–4041. 10.1121/1.3006382 19206825

[B59] Trehub S. E. (2000). “Human processing predispositions and musical universals,” In *The Origins of Music*, eds Wallin N. L. Merker B. & Brown S. (Cambridge, MA: The MIT Press), 427–448.

[B60] Trehub S. E. Unyk A. M. Trainor L. J. (1993). Maternal singing in cross-cultural perspective. *Infant Behav. Dev.* 16 285–295. 10.1016/0163-6383(93)80036-8

[B61] Trevarthen C. (2002). “Origins of musical identity: evidence from infancy for musical social awareness,” in *Musical Identities*, (Macdonald R. A. R. Hargreaves D. J. Miell D.), 21–38.

[B62] Uzgiris I. (1972). “Patterns of vocal and gestural imitation in infants,” in *Determinants of Behavioral Development*, eds Monks F. J. Hartup W. de Witt J. (New York, NY: Academic Press), 467–471. 10.1016/b978-0-12-504750-0.50041-7

[B63] Uzgiris I. (1999). “Imitation as activity: its developmental aspect,” in *Imitation in Infancy*, eds Nadel J. Butterworth G. (Cambridge: Cambridge University Press), 186–206.

[B64] Varela F. J. Thompson E. Rosch E. (2016). *The Embodied Mind: Cognitive Science and Human Experience.* Cambridge, MA: MIT press.

[B65] Wiltermuth S. S. Heath C. (2009). Synchrony and cooperation. *Psychol. Sci.* 20 1–5. 10.1111/j.1467-9280.2008.02253.x 19152536

[B66] Zentner M. Eerola T. (2010). Rhythmic engagement with music in infancy. *Proc. Natl. Acad. Sci.* 107 5768–5773. 10.1073/pnas.1000121107 20231438 PMC2851927

